# Variations in Amino Acid and Protein Profiles in White versus Brown Teff (*Eragrostis Tef*) Seeds, and Effect of Extraction Methods on Protein Yields

**DOI:** 10.3390/foods8060202

**Published:** 2019-06-11

**Authors:** Yoseph Asmelash Gebru, Jun Hyun-II, Kim Young-Soo, Kim Myung-Kon, Kim Kwang-Pyo

**Affiliations:** Department of Food Science and Technology, College of Agriculture and Life Sciences, Chonbuk National University, Jeonju, Jeollabuk-do 561-756, Korea; holden623@naver.com (Y.A.G.); dnrkrk@hanmail.net (J.H.-I.); ykim@jbnu.ac.kr (K.Y.-S.); kmyuko@jbnu.ac.kr (K.M.-K.)

**Keywords:** protein fractionation, white teff, brown teff, amino acid profile, seed storage proteins, essential amino acids

## Abstract

Data on variations in amino acid compositions and protein profiles among white and brown teff, a grain of growing interest, is either limited or contradicting at the moment. In this study, three white (Addis-W, Mekel-W and Debre-W) and three brown (Addis-B, Mekel-B and Debre-B) teff seed samples were used for whole flour amino acid analysis and protein fractionation with three different methods. White and brown seed types showed different physical changes during protein extraction. Brown teff displayed higher essential amino acid content than white with lysine present in high concentration in both seed types. Extraction with tert-butanol increased prolamin yields in teff compared to ethanol. The major protein fraction in teff was glutelin with white teff containing higher glutelin proportion than brown. Sodium Dodecyl Sulfate Gel Electrophoresis (SDS-PAGE) analysis revealed clear genetic variability between white and brown teff seed types.

## 1. Introduction

Teff (*eragrostis tef*) is a small stress tolerant grain (about 0.7% of mass of wheat grain) originally from Ethiopia [1]. Its seed color is either white or very deep reddish brown. In Ethiopia, the second most populous country in Africa, it covers the largest share of area of cereal cultivation with 2.6 million hectares and is a staple food for 80% of the population [2]. Currently, around 200 million people in Ethiopia, Eritrea, Europe and North America consume teff products daily and it is being produced in the USA, Canada, South Africa, Australia and Switzerland [3]. This global demand is a result of its gluten-free nature, high level of essential amino acids (EAA), high mineral content, low glycemic index (GI), high crude fiber content, longer shelf life and slow staling of its bread products compared to wheat, sorghum, rice, barley and maize [4,5,6].

It is well established that the nutritional and food product qualities of pseudocereals such as amaranth and quinoa as well as common grains are mainly attributed to the physiological functions and food processing characteristics of their seed storage proteins (SSPs) [7], and, more importantly, EAA contents. Amaranth contains 17–19% protein and quinoa and buckwheat were found to be rich in essential amino acids, specially lysine [8,9]. The major proteins in amaranth are albumins (33%) followed by glutelins (30%) and prolamins (3%) [10]. unlike proteins from wheat, rice, maize and barley, pseudocereal proteins do not contain allergens and lysine content of amaranth was found to be twice that of wheat and three times that of maize [11]. On the other hand, quinoa and amaranth flour dough showed preferable rheological properties such as an increase in water absorption and reduction of specific volume compared to wheat flour dough [12], due to higher solubility of their proteins. However, most pseudocereals are usually bushy and slow-growing plants that cannot be harvested as fast as common crops. Teff, a stress tolerant, fast growing grain with similar nutritional qualities, has been recently renewing global interest in pseudocereals.

While it is also rich in protein (12.8–20.9%) and EAA contents (~37%) [13,14], limited and sometimes contradicting data are available on the protein and amino acid profiles of Teff. In terms of SSPs, some literature reported glutelin fraction (45%) as a major protein, followed by albumin (37%) and prolamins (12%) [5], while others claimed albumins to be a major protein fraction in white seed types [15]. More recent papers reported prolamins as a major protein fraction with 40% [4,6]. One study reported that the dominant amino acid in teff is glutamic acid+glutamine (21.8 g/16 g N) followed by alanine (10.1 g/16 g N) and leucine (8.5 g/16 g N) [5]. Besides the few studies on amino acid analysis of crude proteins and distribution of protein fractions, no one has attempted to explore the difference between the white and brown seed types from different regions regarding such profiles. 

In this study, we collected six teff samples from 3 different regions in Ethiopia (white and brown samples from each region) and analyzed their amino acid compositions, which allowed, for the first time, comparative nutritional qualities among teff seed types. In addition, we compared three different protein extraction methods to explain the previously contradicting results on major protein fraction in Teff. Further analysis of differently fractionated proteins on SDS-PAGE showed significant difference in protein banding patterns suggesting possible quality or functional differences among teff seed types.

## 2. Materials and Methods

### 2.1. Preparation of Plant Materials

Teff seed samples harvested during 2017 harvest season were purchased from local markets in Addis Ababa (Central Ethiopia; white called Addis-W and brown called Addis-B), Mekelle (Northern Ethiopia; Mekel-W and Mekel-B) and Debremarkos (Western Ethiopia; Debre-W and Debre-B), and brought to South Korea with proper packaging. Whole grains were finely ground using a roll mill (Single type stainless roller, Shinpoong Eng. Ltd., Gwangju, Gyeonggi, Korea) with 0 mm gap between the rollers and 4 smashes. The flour was again sieved through a 1mm mesh sieve and stored at 4 °C until use. 

### 2.2. Chemicals and Reagents

All reagents and buffers were of analytical grade and purchased from Sigma-Aldrich (St. Louis, MO, USA) unless indicated otherwise. 

### 2.3. Amino Acid Analysis

Amino acid compositions of flour samples were determined according to a modified method of Spackman et al. [16]. For hydrolysis, 0.5 g of powdered seed samples were dissolved in 6 N HCl solution and heated for 24 h at 110 °C. The solution was evaporated under reduced pressure using a rotary evaporator (EYELA Rotary vacuum evaporator N-N SERIES, Tokyo Ridadidai Co. Ltd, Tokyo, Japan) and the residual solid was dissolved in sample dilution buffer (pH 2.2). The solution was filtered through a 0.45 µm membrane (ADVANTEC Toyo Roshi Kaisha, Japan). Amino acid analysis was performed using a Sykam (Sykam Co., Darmstadt, Germany) S7130 amino acid reagent organizer, S5200 sample injector and S2100 solvent delivery system with a cation separation column LCA K06/NA (4.6 mm × 250 mm). The flow rates of mobile phase and ninhydrin were 0.45 mL/min and 0.4 mL/min, respectively. Due to the acid hydrolysis, glutamine and asparagine were determined with glutamic acid and aspartic acid, respectively, and tryptophan was degraded and not detected. 

### 2.4. Fractionation of Teff Proteins

Three types of methods were used to sequentially extract four fractions of proteins (albumins, globulins, prolamins and glutelins) from teff seed flour. Method 1 was conducted based on a modified protein fractionation method described by Ayoni et al. [17]. Briefly, albumins were extracted with deionized water on flour to solvent ratio of 1:10 (*w/v*) three times by shaking for 1 h each time and a fourth time for 30 minutes. Each extract was centrifuged at 6000× *g* for 10 min at 4 °C to obtain a clear supernatant, and all supernatants were combined as albumin fraction. The residue after albumin extraction was used to extract globulins using 1.25 M NaCl in a similar procedure for albumins. After washing the residue from the globulin extraction 2 times for 1 h with deionized water to remove salt, prolamins were extracted from the residue using 70% ethanol. Again, after washing with deionized water the same way as above to remove the alcohol, glutelins were extracted with 0.075 M NaOH in similar steps as above. 

Method 2 was done according to a modified version of a method described by Wallace et al. [18]. Albumins and globulins were extracted exactly the same as in method 1. Prolamins were extracted using 70% ethanol containing 5% β-merkaptoethanol (βME) at RT (23 °C) 3 times for 1 h and a fourth extraction done overnight. Glutelins were extracted with 0.075 M NaOH containing 5% βME and 1% SDS the same way as prolamins in this method. 

Method 3 was performed using a modified method by Tylor et al. [19]. Albumins and globulins were extracted exactly the same was as in Method 1 and 2. Prolamins were extracted using 60% tert-butanol containing 0.05% DTT at 23 °C and the fourth time extraction was done overnight. Similarly glutelins were extracted with 0.075M NaOH containing 0.05% DTT at 23 °C. 

Each supernatant was filtered through a 0.45 µm membrane (ADVANTEC Toyo Roshi Kaisha, Japan) to remove insoluble particles and then dialyzed against distilled water with a 3.5 kDa MW cut of dialysis membrane (Spectrum Laboratories, Rancho Dominguez, CA, USA) with distilled water for 24 hours and 4 changes of water. Protein samples were freeze-dried (MCFD8512; Ilshinbiobase Co., Ltd., Gyeonggi, Korea) and stored at −20 °C until used for SDS-PAGE analysis.

### 2.5. Determination of Protein Concentrations

Protein concentrations were determined as described by Bradford et al. using bovine serum albumin (BSA) as a standard [20]. Protein samples were diluted to fit the absorption range of the standard concentrations to a final volume of 100 µL and mixed with 900 µL of Bradford reagent (Sigma, B6916). After vigorous mixing and incubating at room temperature for 10 minutes, absorbance was measured at 595 nm using UV-visible spectrophotometer (Biochrom–Libra S22, Cambridge, UK).

### 2.6. Sodium Dodecyl Sulfate Gel Electrophoresis (SDS-PAGE)

To determine the molecular weight (MW) distribution of each protein fraction, SDS-PAGE was conducted as described by Laemmli [21]. Extracted samples were filtered through a 0.45 µm membrane, dialyzed against distilled water with a 3.5 kDa MW cut of dialysis membrane and freeze dried. Premixed polyacrylamide gel kit was purchased from Bio-Rad (TGX™ FastCast™ Acrylamide Solutions, Bio-Rad Corporations, Hercules, CA, USA). Protein fractions were run in a 5% stacking gel (*w/v* polyacrylamide) and 15% separating gel. 17 µg of protein was loaded per well and run on AE-6531 PAGERUN mini slab electrophoresis system (ATTO Corporation, Tokyo, Japan) for 2 hours at 40 mA. Protein bands were then visualized after staining the gel with coomassie brilliant blue R-250 and MW of prominent bands were estimated by comparing to the Precision Plus Protein Standard (Bio-Rad Laboratories, Hercules, CA, USA). 

### 2.7. Statistical Analysis

All experiments were conducted in triplicate and the results were represented as mean ± standard deviation (SD). One way ANOVA analysis of the mean and variance of the extraction yields was done using Graph pad prism 5 (GraphPad Software, San Diego, CA, USA) to compare the efficiency of methods and distribution of protein fractions and amino acids in white versus brown teff. Differences with *p* < 0.05 were considered significant.

## 3. Results

### 3.1. Physical Characteristics of Teff Seeds

**Figure 1:**
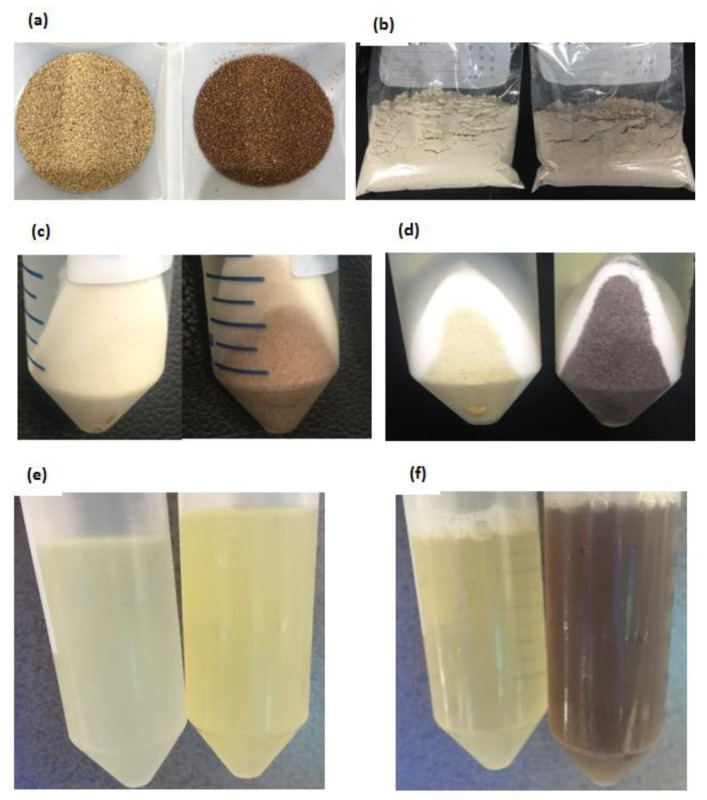
Physical appearances of Mekel-W and Mekel-B during sample preparation. (**a**) Seeds (left to right; Mekel-W, Mekel-B), (**b**) Teff seed flour (left to right; Mekel-W, Mekel-B), (**c**) Residue before ethanol extraction (left to right; Mekel-W, Mekel-B), (**d**) Residue after ethanol extraction (left to right; Mekel-W, Mekel-B), (**e**) tert-butanol extract supernatants (left to right; Mekel-W, Mekel-B), (**f**) NaOH extract supernatants (left to right; Mekel-W, Mekel-B).

All six teff seed samples were ground and used for three different protein fractionation methods, during which color changes in residues and supernatants were monitored. The pictorial data from Mekel-W and Mekel-B during Method 3 extraction procedures were selected as representatives because the same results were observed for all samples (See below). The two seed types have indubitably different colors with linen white and brown colors, respectively (Figure 1a,b). However, the difference in color became less pure when finely ground (Figure 1c,d).

No change in color of residue was observed after albumins (water extraction) or globulins (NaCl extraction) were removed while white teff residue became more elastic and viscous than brown. After alcohol extraction for prolamin fraction, however, the color of the residue of brown teff flour changed from reddish brown to a dark umber color while the white type remained same color (Figure 1e,f). The supernatants of water and salt extractions of both seed types were colorless, while the alcohol extract supernatants of white and brown teff flours were of light and yellowish color, respectively (Figure 1g). After alkaline (0.075M NaOH) extraction, the color of the supernatant was a dark brown or amber color (Figure 1h).

### 3.2. Amino Acid Compositions

**Figure 2:**
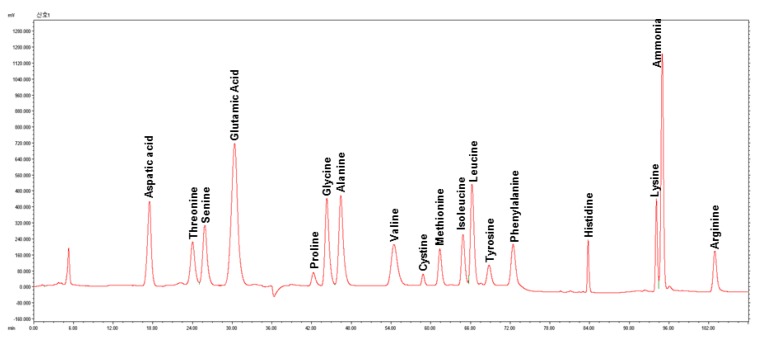
Representative chromatogram showing amino acid profile of teff seed flour (Addis-W). Y-axis is intensity (mAu); X-axis is retention time (min).

Six whole seed flour samples (Addis-W, Addis-B, Mekel-W, Mekel-B, Debre-W and Debre-B) were used for total amino acid analysis and amount of 17 amino acids were determined (Table 1). A representative HPLC chromatogram for amino acid profile of teff seed flour is also shown in Figure 2. The total amino acid contents of all brown teff samples were higher than that of white. No clear distinction in total amino acid contents among the samples of same seed color was observed. The contents of individual amino acids were highly correlated to their total amino acid contents and generally higher in brown teff than that of white teff. With regard to the ratio of each of the amino acids, glutamic acid and glutamine together were the dominant amino acids in all samples, accounting for an average of 26.35% of total amino acid contents followed by aspartic acid and asparagine with average ratio of 9.16%.

The ratio of individual amino acids within each sample showed no significant difference among all 6 samples with the exception of histidine which showed higher proportion in Debre-B (6.96%) than others, and lysine which was higher in Debre-W (9.37%) than others. In addition, arginine was found to be in higher proportion in white teff than in brown, unlike the other amino acids in which their ratios were consistently similar in all samples of both seed types (Table 1). 

The total essential amino acid (EAA) contents of brown teff were higher than white teff. Generally, there was no significant difference in EAA content among the samples of same seed color and ratio among all samples. An exception was Debre-B which showed a notably higher total EAA content (104.50 mg/g flour dry weight) than the other brown teff samples (90.34 and 84.45 for Addis-B and Mekel-B respectively). The ratio of EAA in this particular sample was 46.60% which is far from the average (40.40 and 40.90 for white and brown, respectively). 

Among the individual EAAs, lysine (average of 12.82 mg/g of flour dry weight) was at the highest concentration in white teff, followed by leucine (11.74), isoleucine (9.34), phenylalanine (8.52), threonine (7.05), valine (6.02), methionine (4.52) and histidine (3.02). In brown teff the highest amount was leucine (17.51), followed by lysine (15.12), isoleucine (14.06), phenylalanine (12.52), threonine (10.40), valine (8.73), histidine (8.06) and methionine (6.70). 

### 3.3. Effect of Extraction Methods on the Yield of TSSP

The yields of Teff seed storage protein fraction (TSSPF) prepared by three methods with different extraction solvent compositions were compared and are shown in Table 2.

In Method 1, among Addis Ababa and Debremarkos samples, amounts of total TSSP yields (6.34–7.05 g/100 g flour) and each TSSPF (1.82−2.97 for Albumins, 0.29–0.52 for globulins, 0.1–0.38 for prolamins, 3.19–4.27 g/100 g for glutelins) were very similar regardless of seed colors. On the other hand, significantly lower amounts of total TSSP (4.50 and 3.94 for Mekel-W and Mekel-B, respectively) were extracted from Mekelle samples. In all the samples, glutelin was the most prominent fraction followed by albumins and globulin (or prolamins). In this method, there was no difference in the total protein yield between white and brown seed types. However, the average ratio of glutelins proportion in white teff (57.11%) was higher than that of in brown teff (48.88). There was no significant difference in other fractions between white and brown. 

In Method 2, higher amounts of total TSSP from all the samples (7.16–7.87 g/100 g for Addis Ababa and Debremarkos samples which is significantly higher than 4.96–5.26 g/100 g for Mekelle samples) were extracted compared to Method 1. This was mainly due to the increase in prolamin and glutelin yields. The most notable difference compared to Method 1 was a more profound increase in prolamin fraction, leading to significant increase especially for Mekel-B sample (0.24–1.23 g/100 g) in fractional yield for prolamin. Nevertheless, the most prominent fraction is Glutelin followed by albumins, prolamins and globulins. Similarly, as in Method 1, there was no difference in the total protein yield between white and brown teff. The average ratio of glutelins in white teff (53.68%) was higher than that of in brown teff (45.44%). On the other hand, the average prolamin content (16.83%) in brown teff was higher than that of white (8.3%). Albumin and globulin remained similar. 

In Method 3, most efficient extractions of total TSSP (8.20–9.37 g/100 g for Addis Ababa and Debremarkos samples which is significantly higher than 6.55–6.69 for Mekelle samples) from all the samples were observed compared to the others. Notably, in all the samples, significantly higher (*p* < 0.0001) increases in prolamin fraction yields were observed, even making it to a change in order of magnitude (Mekel-B; for Method 1 vs. Method 3). Among all samples, Mekel-B had significantly higher (*p* < 0.05) prolamin content (2.24 g/100 g or 34.16%) than other samples. Glutelins were major proteins followed by albumins, prolamins and globulins except in Mekelle-B where prolamins held the second place. As in Methods 1 & 2, there was no difference in the total protein yield between white and brown teff. The average ratio of glutelins in white (46.62%) was also higher than that of the brown teff (39.1%). The average prolamin content in brown teff (29.39%) was higher than that of white (21.06%). Albumin and globulin remained similar among the two seed types.

### 3.4. Molecular Weight Distribution of Teff Proteins

**Figure 3:**
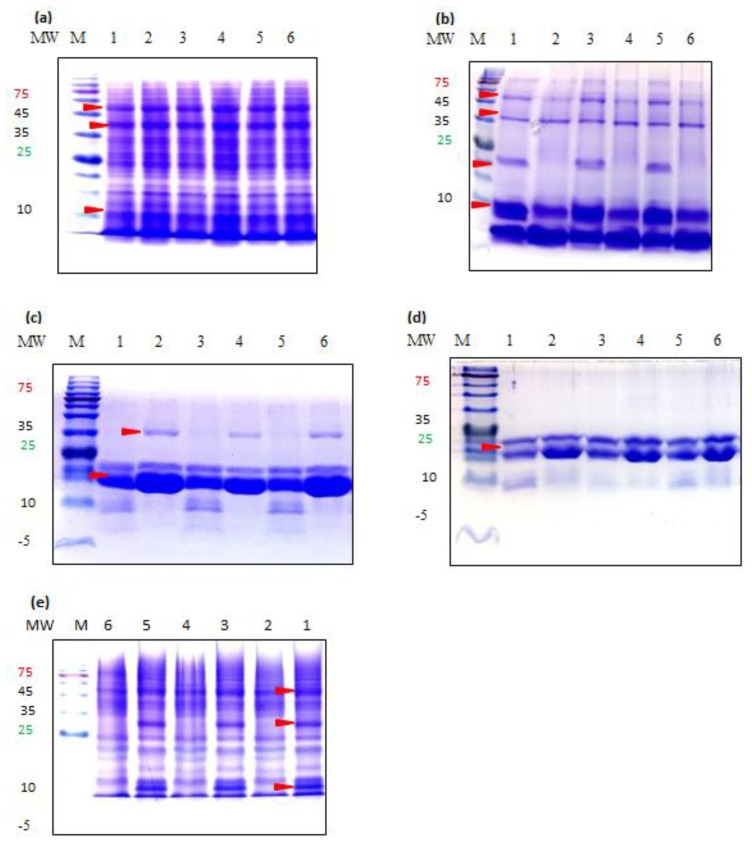
Sodium Dodecyl Sulfate Gel Electrophoresis band patterns of teff protein fractions resolved on a 15% gel under reducing conditions. (**a**) Albumins, (**b**) Globulins, (**c**) Prolamins Method 2, (**d**) Prolamins Method 3, (**e**) Glutelins. MW, molecular weight (kilodaltons); M, marker; Lane 1, Addis-W; Lane 2, Addis-B; Lane 3, Mekel-W; Lane 4, Mekel-B; Lane 5, Debre-W; Lane 6, Debre-B.

Protein fractions extracted with Method 3 are shown for SDS-PAGE analysis as, in general, very similar protein patterns were observed for the same sample extracted with different methods (Figure 3). One exception was prolamin fraction which showed notable difference between Method 3 and the other two methods (See below). 

Albumin fraction was resolved to show no variation between white and brown types of different samples (Figure 3a). The highest number of polypeptides was observed among other fractions with MW ranging from 10–100 kDa. The three most prominent bands were approximately at 10, 53 and 67 kDa in size.

Proteins in globulin fraction were characterized by the low MW proteins being dominant. Regardless of different sample regions, the same banding patterns were observed among the same seed color types, but a clear difference was found between white and brown types (Figure 3b). In white seed type, the fraction included 5 subunits approximately at 6, 9, 22, 37.5 and 52.5 kDa. In the case of brown type, all bands were detected at same locations with similar intensity as in white type with the exception of the 22 kDa subunit which was completely absent.

Proteins in Prolamin fraction were characterized by a high expression level of low MW proteins. In Methods 1 and 2, regardless of different samples regions, four bands approximately at 9.5, 11, 19.5 and 23 kDa for white type and three bands at 19.5, 23 and 35 kDa for brown type were detected (Figure 3c). In Method 3, only the 19.5 and 23 kDa bands were detected in similar patterns in both seed types (Figure 3d). The 23 kDa band which formed a very intense band in both seed types had significantly higher intensity in brown teff than in white. 

Proteins in Glutelin fractions were also diverse, as in albumin fraction, with molecular sizes ranging from approximately 7 to 100 kDa (Figure 3d). Again, similar banding patterns were observed among the samples of different regions, but clear difference was found between seed color types. Most notable difference was the presence of 9 and 30 kDa bands in brown type which are absent in white.

## 4. Discussions

### 4.1. Physical Characteristics

A less visible difference is observed in the flour colors of white and brown teff (Figure 1c,d) compared to the difference in their respective seed colors (Figure 1a,b), indicating that the pigmenting compounds of brown teff are mainly accumulated on the grain pericarp. Previously, it was shown that the endosperm of both seed types have similar composition which is predominantly composed of starch, while the pericarp of brown teff contains various compounds that give the seed its distinct color [22]. 

We also found the changes in the colors of the residue to deep umber (Figure 1e,f) and of the supernatant to light yellowish color of brown teff after alcohol extraction (Figure 1g). A recent study found that while an unusually high level of flavones are present in both white and brown teff, tannins are present as procyanidin (condensed tannins) form only in the brown seed [22]. The fact found in our study that the color of the supernatant turned dark umber (Figure 1h) in the NaOH extract may also suggest that the insoluble tannins are partially soluble in alkaline solution, which is similar to previous studies [23,24]. Further explorations on the ethanol extract and the procyanidins in brown teff may reveal a variety of bioactive compounds accumulated on teff bran and discover more compounds responsible for the color.

### 4.2. Amino Acid Compositions

It is well accepted that the dietary value of SSPs is measured depending on the amino acid composition [25]. In this study, we found that the amino acid compositions of teff were very similar among the same seed color types (white or brown) from different regions while total amino acid content of brown teff was found to be significantly higher (*p* < 0.05) than the white one. Altogether, the total amino acid content of teff (227.74 and 154.87 mg/g flour for brown and white teff respectively) is higher than amaranth (140.1) and quinoa (113.9) [26] which can be a good reason to attract interest on teff research together with these pseudocereals.

Despite the importance of total protein contents, essential amino acid content is more crucial characteristic when assessing the dietary quality of grains. In this study, the total amount of essential amino acids in brown teff was considerably higher than that of white seed type, while the overall ratio of essential amino acids relative to the remaining amino acids was similar in both white and brown (40.4% and 40.88% respectively). Compared to other grains such as wheat (41.5) [27], barely (21.8), maize (25.6) [28], rice 37.8 mg/g of flour [29] etc., Teff seed flour is relatively rich in EAA (62.5–104.5 mg/g of flour) with a well-balanced concentration (Table 1). EAA content and balance has very important value in common cereals which are sometimes the sole source of nitrogen in the developing world.

Lysine, an essential amino acid which exists in a limited amount in other cereals was observed to be in higher concentration (~12–16 mg/g of flour) in teff. This is much higher content compared to the pseudocereals amaranth (7) and quinoa (8.3 mg/g flour) [26]. The fact that lysine exists in limited content in these common cereals which are the staple foods for most of world population is a big challenge in protein diet [30]. Therefore such high lysine content in teff can be considered as a key factor in introducing new grains with high nutritional value.

As shown in the protein fractionation section, the total yield of protein extracts did not show significant difference between white and brown seeds (in spite of significant difference between different region samples of the same color, See below) (Table 2). However the concentration of total amino acids was found to be significantly different between white and brown types, brown teff being higher than white one. This shows that the content of amino acids are independent of the total amount of extracted protein fractions in the seed flour, which was also reported for the pseudocereal quinoa [31]. The possible reason for such miscorrelation in teff is suggested to be the interaction between the insoluble tannins and proteins in brown teff that may have caused insolubility during extraction.

Overall, the higher concentration of total and essential amino acids in brown teff can be considered as its nutritional advantage over the white one. To the best of our knowledge, this is the first study to present an organized comparison of amino acid profiles between white and brown teff seeds and superiority of brown teff.

### 4.3. Effect of Extraction Methods on the Yield of TSSP

SSPs are not only nutritionally important but also influence the utilization of grains in food processing. Their characteristics are more important in grains such as wheat and teff which are consumed after processing into various kinds of foods. This called for exploring the less characterized TSSPs to review the existing contradicting data on their fractions.

In the case of prolamins, Method 3 resulted in significantly higher (*p* < 0.001) yield followed by Method 2 and then Method 1 (Table 2). This showed that tert-butanol was much more effective than ethanol in extracting alcohol soluble proteins from cereals. Tert-butanol has been designated as a natural gift for protein isolation for its advantage in stabilizing protein structures during extraction. This might be attributed to its larger size that can hinder it from accessing the interior of the protein which stabilizes the protein instead of denaturing. It also inhibits enzyme activities and protein-protein interactions minimizing formation of artifacts and resulting in higher extraction yield [31,32]. Based on this, it can be assumed that the increase in prolamin yield is due to an overall improved stability and less proteolysis of the protein fraction.

Method 2 also significantly (*p* < 0.05) increased prolamin yield compared with Method 1 which is attributed to the reducing agent βME (Table 2). βME is a strong reducing agents that cleave disulfide crosslinks in proteins and also inhibit oxidation of free sulfhydryl residues. The increased prolamin yield in Method 2 (average 0.79 g/100 g flour) from Method 1 (0.09) indicate that teff prolamines are prone to oxidative damage and can be effectively protected by reducing agents during extraction. This protein fraction has been reported to form disulfide bonds during extraction and reducing agents effectively enhanced their extraction yield by preventing the formation of disulfide bonds [33]. Here, it can be assumed that the increase in prolamin yield is due to an increase in overall solubility and a decrease in the oxidation damage of the fraction.

In the case of glutelin fraction, there was only a slight difference in yield between Method 1 and Method 3 but not between Method 1 and 2 (Table 2). In grains such as maize, glutelin has shown to be highly insoluble in the most potent protein dissociating solvents [34]. However, with reduction of disulfide bonds by βME in the extraction solution, it was possible to increase its yield [35]. In this study, glutelin fraction was extracted using 0.075 M NaOH with or without the reducing agents βME and DTT. Even though DTT showed a relatively better effect than βME similar to the previous study [36], there was no significant difference compared to Method 1 (no reducing agent). This indicates that the glutelin fraction in a gluten free teff seed is relatively readily soluble compared to the glutelin in maize (a high gluten grain). It has been well established that glutelin is the major component of gluten and the gluten level in cereals is linearly related to the percentage of disulfide bonds in the glutelin fraction [37]. Therefore, the gluten free nature of teff and the no effect observed with or without reducing agents during extraction could be attributed to a less percentage of disulfide crosslinks in teff glutelin proteins.

In method 1 the ratio was glutelins > albumins > globulins > prolamins in which globulins and prolamins comprised only a small portion. The distribution order in method 2 and 3 was glutelins > albumins > prolamins > globulins. However, the relative proportion of prolamins and albumins in method 3 was almost similar while their variation gap was wider in method 2. This is partially in agreement with [5,38] who reported glutelins as major proteins (45%) followed by albumins (37%) and prolamins (12%), yet it is in contrast with a result reported by Abdul-Rasaq et al. [4] where prolamin was the major fraction accounting for 40% of the total protein in teff and Zhang et al. [6] which suggested prolamin was the major protein after analyzing the amino acid composition of teff. It is common to obtain different results of protein proportions with different extraction methods and samples from growth environments. Even though we used the same solvent as Abdul-Rasaq et al. [4], the different result might be due to different samples. In one of our samples (Mekel-B) prolamin ratio (34.16%) was almost similar with glutelin (37.71%).

In Method 3, white teff was found to have significantly higher glutelin content (46.62%) than brown (39.1%) on average. Previous studies have confirmed that glutelin together with prolamin plays an important role in bread making characteristics in wheat and other grains [39]. We observed that the rheology of the residue of white teff before glutelin extraction was much more viscous and elastic compared to brown. Therefore, the higher proportion of glutelins in white teff may have a big influence on functional properties including baking performance and dough rheology.

On the other hand, the average prolamin content (29.39%) of brown teff in Method 3 was higher than that of white teff (21.06%). It has been proven that prolamins play a great role in aggregation of protein bodies (PBs) in teff and maize endosperms as examined by transmission electron microscopy (TEM) [6]. Therefore, the higher expression of prolamins in brown teff may also influence the functional properties in food processing. Similarly, prolamins with their distinctive amino acid compositions can alter the overall proportion of essential amino acids in brown teff thereby bringing nutritional difference. In this study we also proved that brown teff contains significantly higher essential amino acids content than white teff (Table 1). To have a deeper understanding of the difference in physical and nutritional properties between white and brown varieties, a more detailed study on teff prolamins is required.

While the total protein yields of Addis Ababa and Debremarkos samples were somehow similar (Table 1), the samples from Mekelle showed significantly lower total protein yield. It is common to observe variations in protein content across locations while amino acids are stable [40]. Therefore, the difference might have been caused by different environmental conditions during plant development.

### 4.4. SDS-PAGE

SDS PAGE analysis was conducted to examine any variation of polypeptide patterns. Despite different total amounts of protein fractions among the samples from different regions (Table 2), no quantitative or qualitative variation was observed among the same seed types from different regions. However, clearly visible differences in band numbers and band quantities were observed between white and brown seed types, except for the albumin fraction which showed similar patterns. Our prolamin patterns are in partial agreement with Abdul Rasak et al who detected the 19.5 and 23 kDa subunits for both white and brown teff at similar MW [4]. Zhang et al. also reported similar results for brown teff with two most predominant bands detected at 19 & 22 kDa [6]. We could not find previous SDS PAGE patterns of TSSPFs in the literature except for prolamins.

Our study discovered significant variation in protein profiles between white and brown teff based on their SDS PAGE patterns of globulin, prolamin and glutelin fractions. The SDS PAGE pattern variation between white and brown teff can be used as a tool to study genetic diversities and identification of particular proteins in teff, because SSPs are highly independent of environmental fluctuations [41]. The absence or presence and differences in band intensities can also be regarded as a basis for a possible polymorphism studies in teff since the type and amount of proteins in mature seeds are constant [42]. Further studies will be required for identification of seed type-specific proteins.

## 5. Conclusions

The yellowish color after alcohol extraction of teff flour can be an evidence for the presence of phenolic compounds and flavonoids, and some of the main compounds that give brown teff seed its color are alkaline soluble and are probably insoluble condensed tannins. Regarding nutritional qualities, Teff has superior essential amino acid content and balance compared to wheat, rice, barely and maize, with the brown seed having higher amino acid content than the brown. The addition of reducing agents to extraction solvents enhances prolamin yield during teff protein fractionation. Tert-butanol is more efficient than ethanol for extraction of teff prolamins. Glutelin is a major protein in teff seeds. The extracted glutelin content of white teff is significantly higher than that of brown teff. SDS-PAGE analysis can reveal genetic variability storage proteins among white and brown teff.

## Figures and Tables

**Table 1:** Amino acid composition (mg/g flour dry weight) and ratio of individual amino acid (%) of 6 teff flour samples.

Amino Acid		Samples											
		Addis-W	Ratio (%)	Addis-B	Ratio (%)	Mekel-W	Ratio (%)	Mekel-B	Ratio (%)	Debre-W	Ratio (%)	Debre-B	Ratio (%)
**Essential Amino Acids**	**Thr**	6.73 ± 0.35	4.35	10.76 ± 0.66	4.48	7.53 ± 0.15	4.68	10.45 ± 0.07	4.78	6.90 ± 0.14	4.52	10.00 ± 0.00	4.46
	**Val**	5.60 ± 0.20	3.62	8.63 ± 0.32	3.59	5.66 ± 0.32	3.52	8.35 ± 0.21	3.82	6.80 ± 0.28	4.46	9.20 ± 0.84	4.1
	**Met**	4.10 ± 0.17	2.65	7.16 ± 0.32	2.98	4.66 ± 0.49	2.9	6.15 ± 0.35	2.81	4.80 ± 0	3.15	6.80 ± 0.28	3.03
	**Ile**	9.23 ± 0.05	5.96	14.13 ± 0.11	5.88	9.20 ± 0.45	5.72	13.85 ± 0.63	6.33	9.60 ± 0.00	6.29	14.20 ± 0.28	6.33
	**Leu**	11.40 ± 0.26	7.36	17.53 ± 0.28	7.29	11.73 ± 0.64	7.29	16.70 ± 0.14	7.64	12.10 ± 0.42	7.93	18.30 ± 0.42	8.16
	**Phe**	8.23 ± 0.58	5.31	12.60 ± 0.36	5.24	8.43 ± 0.3	5.24	11.35 ± 0.49	5.19	8.90 ± 0.70	5.83	13.60 ± 0.84	6.07
	**His**	2.96 ± 0.05	1.91	4.43 ± 0.05	1.84	3.13 ± 0.11	1.94	4.15 ± 0.21	1.9	3.10 ± 0.14	2.03	15.60 ± 1.69	6.96
	**Lys**	12.00 ± 0.17	7.75	15.10 ± 0.40	6.28	12.16 ± 0.6	7.56	13.45 ± 0.49	6.15	14.30 ± 0.70	9.37	16.80 ± 0.00	7.49
	**Subtotal**	60.25	38.91	90.34	37.58	62.5	38.85	84.45	38.62	66.5	43.58	104.5	46.6
**Other amino acids**	**Asp**	15.93 ± 0.55	10.29	19.23 ± 0.86	8	15.06 ± 0.75	9.36	18.30 ± 0.98	8.37	15.60 ± 0.84	10.22	19.60 ± 0.00	8.74
	**Ser**	7.16 ± 0.55	4.62	11.06 ± 0.66	4.6	7.86 ± 0.25	4.88	10.90 ± 0.14	4.99	7.10 ± 0.42	4.65	11.20 ± 0.00	5
	**Glu**	38.90 ± 0.34	25.12	75.86 ± 8.25	31.56	43.86 ± 1.78	27.25	63.35 ± 9.26	28.97	33.50 ± 0.98	21.95	52.10 ± 2.68	23.24
	**Pro**	1.86 ± 0.11	1.2	2.96 ± 0.32	1.23	1.96 ± 0.32	1.22	2.95 ± 0.07	1.35	2.10 ± 0.42	1.38	3.40 ± 0.00	1.52
	**Gly**	5.76 ± 0.15	3.72	7.50 ± 0.26	3.12	6.00 ± 0.40	3.73	6.90 ± 0.28	3.16	5.90 ± 0.42	3.87	7.60 ± 0.28	3.39
	**Ala**	7.06 ± 0.15	4.56	10.40 ± 0.26	4.33	7.50 ± 0.51	4.66	12.15 ± 2.75	5.56	9.00 ± 0.28	5.9	10.40 ± 0.00	4.64
	**Cys**	0.63 ± 0.05	0.41	1.13 ± 0.05	0.47	0.63 ± 0.05	0.39	0.85 ± 0.07	0.39	0.4 ± 0.00	0.26	ND	ND
	**Tyr**	3.36 ± 0.05	2.17	5.46 ± 0.05	2.27	3.43 ± 0.15	2.13	5.05 ± 0.07	2.31	ND	ND	3.60 ± 0.00	1.61
	**Arg**	13.96 ± 1.22	9.01	16.43 ± 0.32	6.84	12.13 ± 0.75	7.54	13.75 ± 0.49	6.29	12.5 ± 0.98	8.19	11.80 ± 1.41	5.26
	**Subtotal**	94.62	61.1	150.03	62.42	98.43	61.16	134.2	61.39	86.1	56.42	119.7	53.4
**Total**		154.87 ± 1.01	100	240.37 ± 4.17	100	160.93 ± 7.80	100	218.65 ± 8.27	100	152.6 ± 4.24	100	224.2 ± 14.56	100

Values are mean ± standard deviation of triplicate. (*p* < 0.05). Thr = Threonine; Val = Valine; Met = Methionine; Ile = Isoleucine; Leu = Leucine; Phe = Phenylalanine; His = Histidine; Lys = Lysine; Asp = Aspartic acid; Ser= Serine; Glu = Glutamic acid; Pro = Proline; Gly = Glycine; Ala = Alanine; Cys = Cysteine; Tyr = Tyrosine; Arg = Arginine.

**Table 2:** Effects of different extraction methods on yield of Teff seed storage protein fractions (TSSPF).

Sample	TSSPF	Method 1		Method 2		Method 3	
		Yield (g/100g flour)	Ratio (%)	Yield (g/100g flour)	Ratio (%)	Yield (g/100g flour)	Ratio (%)
**Addis-W**	Albumin	2.13 ± 0.22	31.88	2.13 ± 0.16	29.7	2.06 ± 0.09	23.01
	Globulin	0.38 ± 0.01	5.71	0.38 ± 0.01	5.31	0.36 ± 0.05	3.97
	Prolamin	0.10 ± 0.03 ^a^	1.46	0.43 ± 0.01 ^b^	5.96	1.62 ± 0.18 ^c^	18.09
	Glutelin	4.07 ± 0.30 ^a^	60.94	4.23 ± 0.25 ^a^	59.04	4.93 ± 0.19 ^b^	54.92
	Total	6.69 ± 0.55 ^a^	100	7.16 ± 0.41 ^a^	100	8.97 ± 0.27 ^b^	100
**Addis-B**	Albumin	2.43 ± 0.29	38.37	2.50 ± 0.24	33.97	2.53 ± 0.15	26.98
	Globulin	0.52 ± 0.12	8.28	0.55 ± 0.08	7.44	0.38 ± 0.29	4.08
	Prolamin	0.19 ± 0.03 ^a^	3.02	0.94 ± 0.02 ^b^	12.77	2.37 ± 0.32 ^c^	25.29
	Glutelin	3.19 ± 0.18 ^a^	50.33	3.37 ± 0.22 ^a^	45.81	4.09 ± 0.27 ^b^	43.64
	Total	6.34 ± 0.43 ^a^	100	7.35 ± 0.38 ^b^	100	9.37 ± 0.39 ^c^	100
**Mekel-W**	Albumin	1.88 ± 0.14	41.81	2.01 ± 0.13	38.13	2.10 ± 0.16	31.37
	Globulin	0.37 ± 0.01	8.26	0.26 ± 0.04	5.01	0.30 ± 0.01	4.46
	Prolamin	0.19 ± 0.01 ^a^	4.12	0.68 ± 0.05 ^b^	12.98	1.75 ± 0.11 ^c^	26.22
	Glutelin	2.06 ± 0.08 ^a^	45.81	2.31 ± 0.10 ^bc^	43.88	2.54 ± 0.10 ^c^	37.95
	Total	4.50 ± 0.23 ^a^	100	5.26 ± 0.19 ^b^	100	6.69 ± 0.36 ^c^	100
**Mekel-B**	Albumin	1.53 ± 0.21	38.84	1.26 ± 0.17	25.36	1.71 ± 0.23	26.07
	Globulin	0.17 ± 0.02	4.27	0.14 ± 0.02	2.88	0.13 ± 0.02	2.06
	Prolamin	0.24 ± 0.01 ^a^	6	1.23 ± 0.10 ^b^	24.73	2.24 ± 0.05 ^c^	34.16
	Glutelin	2.01 ± 0.08 ^a^	50.89	2.33 ± 0.07 ^bc^	47.03	2.47 ± 0.10 ^c^	37.71
	Total	3.94 ± 0.16 ^a^	100	4.96 ± 0.32 ^b^	100	6.55 ± 0.21 ^c^	100
**Debre-W**	Albumin	1.82 ± 0.13	27.57	2.36 ± 0.14	30.8	2.42 ± 0.19	29.53
	Globulin	0.29 ± 0.04	4.36	0.39 ± 0.01	5.13	0.38 ± 0.07	4.62
	Prolamin	0.23 ± 0.02 ^a^	3.5	0.46 ± 0.01 ^b^	5.96	1.55 ± 0.28 ^c^	18.87
	Glutelin	4.27 ± 0.20	64.57	4.44 ± 0.14	58.11	3.85 ± 0.18	46.98
	Total	6.61 ± 0.30 ^a^	100	7.65 ± 0.25 ^ab^	100	8.20 ± 0.62 ^b^	100
**Debre-B**	Albumin	2.97 ± 0.27	42.14	2.82 ± 0.21	35.78	2.65 ± 0.16	30.29
	Globulin	0.50 ± 0.10	7.03	0.61 ± 0.08	7.75	0.44 ± 0.07	5.05
	Prolamin	0.38 ± 0.05 ^a^	5.39	1.02 ± 0.03 ^b^	12.98	2.51 ± 0.22 ^c^	28.71
	Glutelin	3.20 ± 0.23	45.43	3.42 ± 0.19	43.48	3.15 ± 0.18	35.95
	Total	7.05 ± 0.53 ^a^	100	7.87 ± 0.35 ^ab^	100	8.75 ± 0.14 ^b^	100

Values are mean ± SD of triplicate. Values with different superscript alphabets within the same row under protein yields are significantly different (*p* < 0.05).
